# Phantom-based radiomics feature test–retest stability analysis on photon-counting detector CT

**DOI:** 10.1007/s00330-023-09460-z

**Published:** 2023-02-21

**Authors:** Alexander Hertel, Hishan Tharmaseelan, Lukas T. Rotkopf, Dominik Nörenberg, Philipp Riffel, Konstantin Nikolaou, Jakob Weiss, Fabian Bamberg, Stefan O. Schoenberg, Matthias F. Froelich, Isabelle Ayx

**Affiliations:** 1 Department of Radiology and Nuclear Medicine, University Medical Center Mannheim, Heidelberg University, Theodor-Kutzer-Ufer 1-3, 68167 Mannheim, Germany; 2 Department of Radiology, German Cancer Research Center, Im Neuenheimer Feld 280, 69120 Heidelberg, Germany; 3 Department of Diagnostic and Interventional Radiology, University of Tübingen, Hoppe-Seyler-Straße 3, 72076 Tübingen, Germany; 4 Department of Diagnostic and Interventional Radiology, Medial Center-University of Freiburg, Hugstetter Str. 55, 79106 Freiburg Im Breisgau, Germany

**Keywords:** Tomography, X-ray computed, Data science, Radiology

## Abstract

**Objectives:**

Radiomics image data analysis offers promising approaches in research but has not been implemented in clinical practice yet, partly due to the instability of many parameters. The aim of this study is to evaluate the stability of radiomics analysis on phantom scans with photon-counting detector CT (PCCT).

**Methods:**

Photon-counting CT scans of organic phantoms consisting of 4 apples, kiwis, limes, and onions each were performed at 10 mAs, 50 mAs, and 100 mAs with 120-kV tube current. The phantoms were segmented semi-automatically and original radiomics parameters were extracted. This was followed by statistical analysis including concordance correlation coefficients (CCC), intraclass correlation coefficients (ICC), as well as random forest (RF) analysis, and cluster analysis to determine the stable and important parameters.

**Results:**

Seventy-three of the 104 (70%) extracted features showed excellent stability with a CCC value > 0.9 when compared in a test and retest analysis, and 68 features (65.4%) were stable compared to the original in a rescan after repositioning. Between the test scans with different mAs values, 78 (75%) features were rated with excellent stability. Eight radiomics features were identified that had an ICC value greater than 0.75 in at least 3 of 4 groups when comparing the different phantoms in a phantom group. In addition, the RF analysis identified many features that are important for distinguishing the phantom groups.

**Conclusion:**

Radiomics analysis using PCCT data provides high feature stability on organic phantoms, which may facilitate the implementation of radiomics analysis likewise in clinical routine.

**Key Points:**

*• Radiomics analysis using photon-counting computed tomography provides high feature stability.*

*• Photon-counting computed tomography may pave the way for implementation of radiomics analysis in clinical routine.*

**Supplementary Information:**

The online version contains supplementary material available at 10.1007/s00330-023-09460-z.

## Introduction

Radiomics analysis is widely used in clinical research due to the rising interest in transforming subjective and qualitative to objective and quantifiable medical image analysis. This pixel-based extraction of features from imaging data promises additional insights beyond the level of information visible to the human eye [1] using dedicated software packages [2]. The further information consists of a vast number of features of the region of interest (ROI), fostering the big data trends in healthcare and creating new possibilities and promises, especially in oncologic imaging [3, 4]. It is evident that radiomics is able to outperform traditional clinical scores [5, 6] and shows promising results in terms of outcome prediction [7, 8], and tumor classification [4, 9, 10]. Besides oncologic imaging, radiomics analysis paved the way for future developments also in cardiovascular imaging by outlining a better risk stratification, e.g., via coronary plaque analysis [11], and defining potential biomarkers for the development of arteriosclerosis [12]. Additionally, potential myocardial diffuse fibrosis could be detected by radiomics analysis [13]. The power of radiomics analyses has also been shown in lung fibrosis [14, 15], kidney stones [16], and COVID pneumonia [17, 18]. Even the ability of a CT-derived radiomics score to predict the added benefit of adjuvant chemotherapy following surgery in patients with non-small cell lung cancer could be demonstrated by using three intratumoral and 10 peritumoral CT radiomics features [19].

Despite these excellent capabilities, radiomics analysis has not been implemented in clinical practice. One of the most important limitations for clinical implementation is the lack of comparability of radiomics analyses. In the past, several studies have demonstrated the influence of various parameters on the stability of radiomics features, including multiple image acquisition parameter settings such as tube voltage, reconstruction kernel, choice of contrast agent and contrast media phases, slice thickness, and the choice of the scanner which impede the reproducibility [20, 21].

Even the choice of segmentation method [22] and feature extraction software can have an influence on radiomics feature stability [23]. For accurate texture analysis, optimal spatial resolution and signal-to-noise ratio have been defined as the two most important image quality factors [24–26]. In the past, these parameters may have been impacted by the indirect conversion of X-ray photons to an electrical signal with an intermediate scintillator-based step.

Through the implementation of photon-counting CT (PCCT), this hurdle could be possibly addressed. This new technology allows direct conversion of the X-ray photons into electric pulses without the need of converting into visible light, as in traditional energy-integrating CT (EICT). Hence, each photon that reaches the detector contributes to the final image. Additionally, a threshold for image noise can be set for each photon, reducing the overall image noise and in total resulting in better spatial resolution, lower beam-hardening artifacts, and better signal-to-noise ratio [27, 28].

Primary studies have shown radiomics feature properties compared between EICT and PCCT [29, 30], but to our knowledge, an organic phantom-based analysis regarding scan and rescan as well as reposition feature stability with mAs variance on PCCT has not been studied. The aim of this study is to analyze feature stability within the organic phantoms as well as the possibility of radiomics-based differentiation between different phantom groups. We hypothesized that given its technical innovation, PCCT may provide improved radiomics feature stability.

## Materials and methods

### Study design

**Fig. 1:**
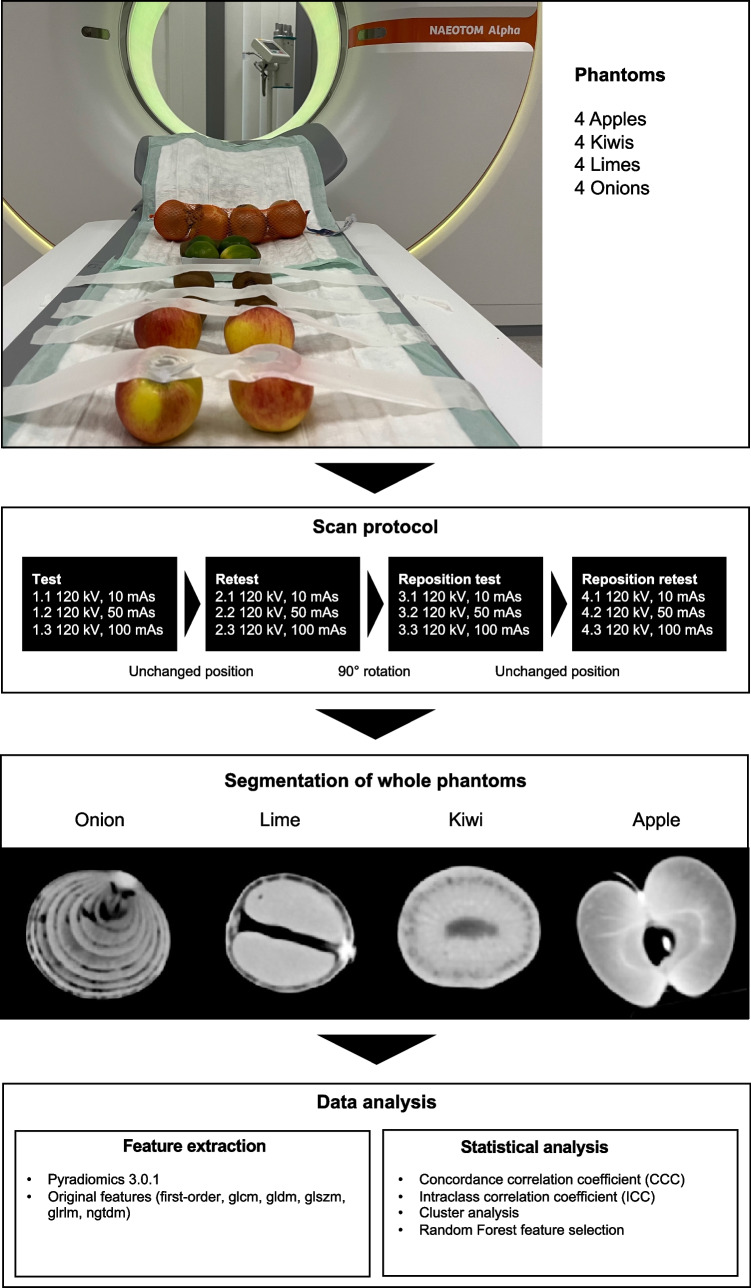
Study flowchart

For this phantom-based single-center study, 16 organic phantoms were included consisting of four apples, four onions, four limes, and four kiwis. The methodological structure of the study is shown in Fig. 1.

### CT imaging

All 16 phantoms were scanned in one session on a first-generation whole-body dual-source PCCT system (NAEOTOM Alpha; Siemens Healthcare GmbH). Underlying scan parameters were a tube voltage of 120 kV and gantry rotation time of 0.25 s. The in-plane resolution of the PCCT is 0.11 mm. A three-level variance was established for mAs, consisting of a scan with 10 mAs, 50 mAs, and 100 mAs. Each of these scans was repeated again immediately afterward without any change in the phantom position or the underlying scan parameters. In the next step, all phantoms were rotated 90° in the same direction (clockwise) and all scans including rescans and mAs variation were repeated (Fig. 1).

### CT imaging analysis

**Fig. 2:**
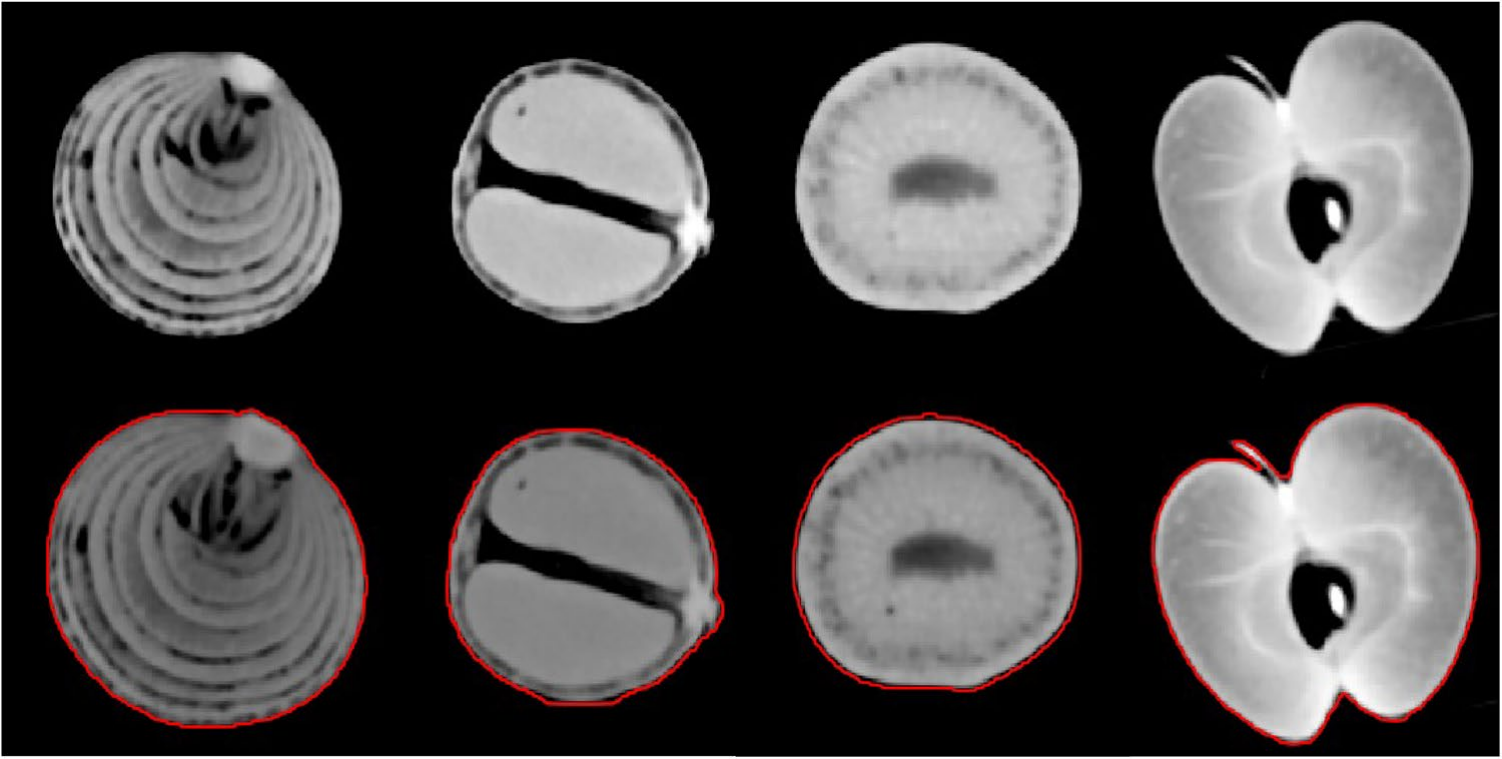
Example segmentation of each phantom group

Axial images of all scans were reconstructed with a slice thickness of 1 mm (increment 1 mm) using a soft tissue kernel (Br40). This data was exported and stored in Digital Imaging and Communications in Medicine (DICOM) file format. DICOM files were converted to Neuroimaging Informatics Technology Initiative (NIFTI) file format for further processing with a dedicated segmentation tool (MITK workbench, version 2021.10) [31]. Segmentation for each organic phantom in each scan was done semi-automatically by a clinical radiologist with 3 years of experience in segmentation. Figure 2 shows an example segmentation as well as the original DICOM images.

### Radiomics feature extraction

Phantom segmentations were further analyzed by radiomics feature extraction using a dedicated imaging biomarker standardization initiative definition–based Python package (PyRadiomics, version 3.0.1.) [2]. For each phantom regarding each scan, first-order features, shape features, and second-order features (in total 104 features), namely gray level co-occurrence matrix (GLCM), gray level dependence matrix (GLDM), gray level size zone matrix (GLSZM), gray level run length matrix (GLRLM), and neighboring gray tone difference matrix (NGTDM), were extracted. The extraction was performed with voxel normalization, resampling to 2 × 2 × 2 mm and rebinning with a fixed bin width of 25 HU. The Chebyshev distance is 1.

### Statistical analysis

All statistical analyses were performed in R (R Core Team 2021) and RStudio (version 1.3.1093) [32].

The statistical analysis was performed in four steps (I to IV):

- In the first step (I), concordance correlation coefficient (CCC) analysis was used to determine the feature stability between test and retest for the original and repositioned phantoms. In the CCC analysis, a default of ≥ 0.9 was defined as a threshold for feature stability in the test-rest setting. This threshold was defined in accordance to Mcbride et al (2005): “A proposal for strength-of-agreement criteria for Lin’s concordance” [33].
- In the second step (II), the feature stability between the scans with different mAs values (10 mAs, 50 mAs, and 100 mAs) was determined by intraclass correlation coefficient (ICC) analysis, for which ICC (3,1) (a fixed set of raters, no average observations) was used as recommended in literature [34]. In the ICC analyses performed, in accordance with Koo and Li, values below 0.5, between 0.5 and 0.75, between 0.75 and 0.9, and above 0.90 were considered suggestive of poor, moderate, good, and excellent stability [35].
- In the next step (III), two different stability tests were performed: In the inter-phantom group stability test, the stability of the radiomics features was compared between two different groups (for example, the group of apples and onions), while in the intra-phantom group stability test, the stability of the individual phantoms within a phantom group (for example, between apples 1 and 4) was tested using ICC analysis. For this ICC analysis, ICC (3,1) was likewise used as explained in step II.
- In step IV, a random forest (RF) analysis was performed with the Boruta algorithm in R [36] to determine the feature importance with respect to the discrimination of the different phantom groups. For each parameter variable, shadow variables are created by permutation by the algorithm. If the real variable is significantly more important than the shadow variable, high importance is assigned to the feature by the algorithm.

In addition, the feature correlations were calculated by Pearson’s correlation coefficients and were visualized in form of heatmaps using the “ComplexHeatmap” package in R.

## Results

### Feature stability analysis

#### Test–retest stability and reposition stability (I)

In the CCC analysis performed (I), 73 features (70% of the extracted features) with excellent stability (CCC value > 0.9) could be identified in the comparison between the test and retest scan (Supplementary Table 1). For non-shape features, 66% of the features still showed excellent stability. All of the extracted shape features showed excellent stability. Percentage-wise, there were more stable first-order features than second-order features: 14 of the 18 extracted first-order features (78%) showed excellent stability with a CCC value above 0.9 while 46 of the 73 extracted second-order features (63%) showed excellent test–retest stability.

In comparison between the original test and the first reposition scan, 68 features showed a CCC value above 0.9 (65.4%).

#### Stability with different mAs values (II)

The ICC analysis in the next evaluation step (II) of the test scans with different mAs values resulted in 78 (75%) features with excellent stability.

#### Inter-phantom group stability (III)

**Fig. 3:**
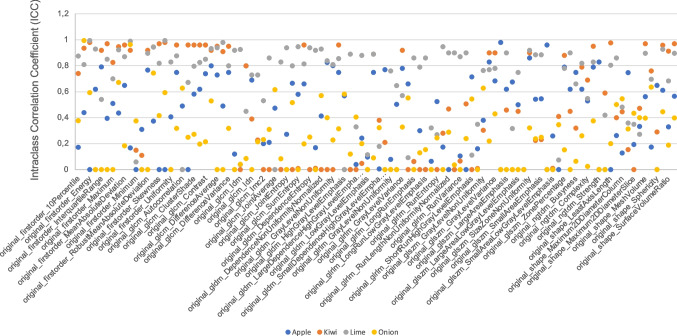
ICC analysis between the individual phantoms of a phantom group

The phantom group is defined by the four fruit/vegetable categories. To analyze the feature stability between the different phantom groups, all apples, onions, kiwis, and limes respectively were included in one group each. To compare the group of, e.g., apples against the group of, e.g., onions, an inter-phantom group stability test was performed. In step (III), the comparison of radiomics stability of individual phantoms within a phantom group resulted in a total of 8 features with an ICC value > 0.75 across at least 3 of the total 4 phantom groups: *original_firstorder_90Percentile*, *original_firstorder_Median*, *original_firstorder_RobustMeanAbsoluteDeviation*, *original_glcm_Correlation*, *original_glszm_GrayLevelNonUniformityNormalized*, *original_glszm_SizeZoneNonUniformity*, *original_glszm_ZonePercentage*, and *original_ngtdm_Contrast* (see Table 1). The results of the ICC analysis are presented in Fig. 3.

**Table 1:** Stable features defined as ICC > 0.75 (analysis of the radiomics stability between the individual phantoms of a phantom group) in at least 3 phantom groups

Feature	Apple	Kiwi	Lime	Onion
original_firstorder_90Percentile	0.44	**0.936**	**0.81**	**0.995**
original_firstorder_Median	0.167	**0.92**	**0.99**	**0.9636**
original_firstorder_RobustMeanAbsoluteDeviation	**0.767**	**0.92**	**0.897**	< 0.001
original_glcm_Correlation	**0.8**	**0.92**	**0.932**	0.529
original_glszm_GrayLevelNonUniformityNormalized	**0.83**	**0.9**	**0.77**	0.22
original_glszm_SizeZoneNonUniformity	**0.86**	**0.9**	**0.92**	0.322
original_glszm_ZonePercentage	**0.79**	**0.88**	**0.781**	0.1535
original_ngtdm_Contrast	**0.79**	**0.95**	**0.83**	0.376

#### Intra-phantom group stability analysis

To investigate the comparative stability of radiomics features within each phantom group, ICC values were compared (Fig. 3). For the intra-phantom group stability analysis, the phantom group was defined by the fruit/vegetable category, meaning one group consisted, for example, of four apples. For apple and onion phantoms, two features reached an ICC value of above 0.9. For the kiwi and lime phantom, this value was reached in 29 and 19 features, respectively.

Table 2 offers an overview of the distribution of stable features by test type and by category.

**Table 2:** Distribution of stable features by test type (test–retest stability, reposition stability, and mAs stability) and by feature category (shape features, first-order features, and second-order features)

	Stable shape features in %	Stable first-order features in %	Stable second-order features in %
Test–retest stability (CCC)	100	77.8	63
Reposition stability (CCC)	85	66.7	61.6
mAs stability (ICC)	100	94.4	65.8

### Feature selection

#### Feature importance (IV)

**Fig. 4:**
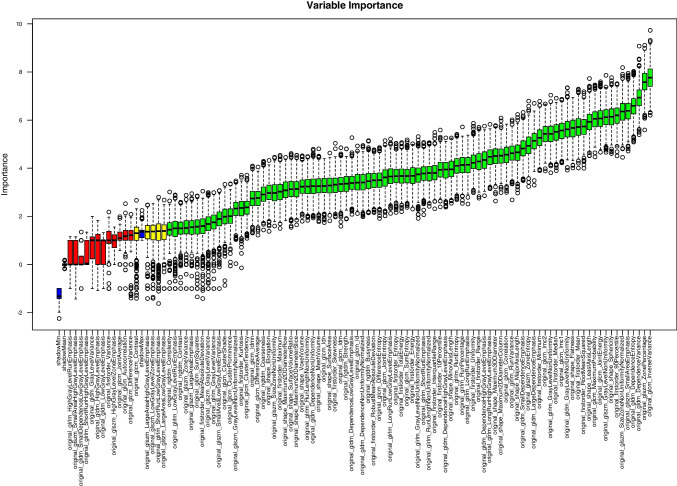
Output of the random forest feature selection ranked by importance using the Boruta algorithm

The performed Boruta random forest analysis (step IV) revealed that 88 of the 104 extracted radiomics features (84.6%) were important for the discrimination between different phantom types. The results are shown as a Boruta plot in Fig. 4.

### Cluster analysis

**Fig. 5:**
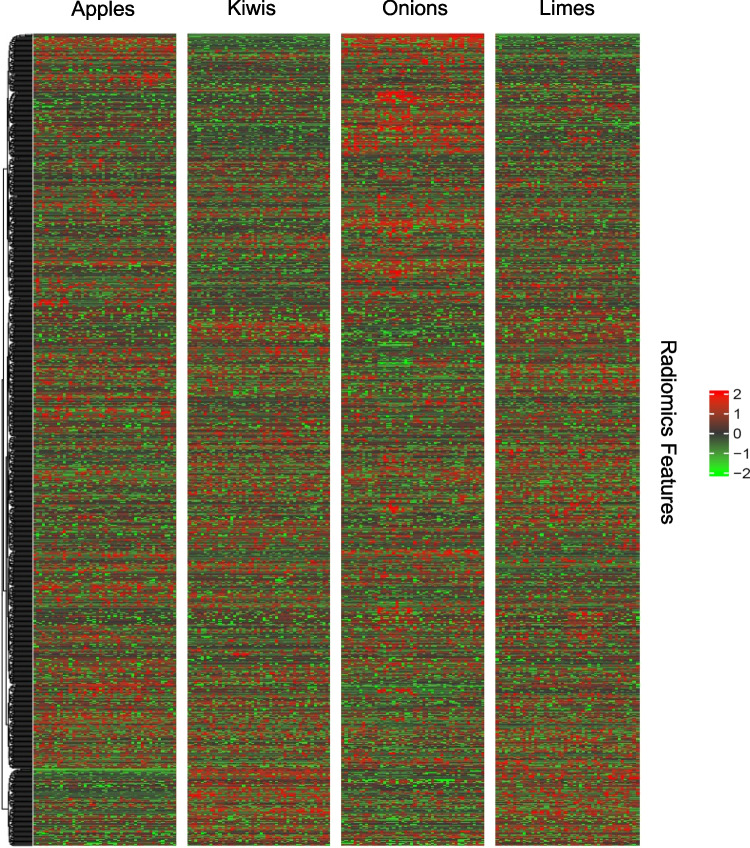
Split by phantom group heatmap of all radiomics parameters

**Fig. 6:**
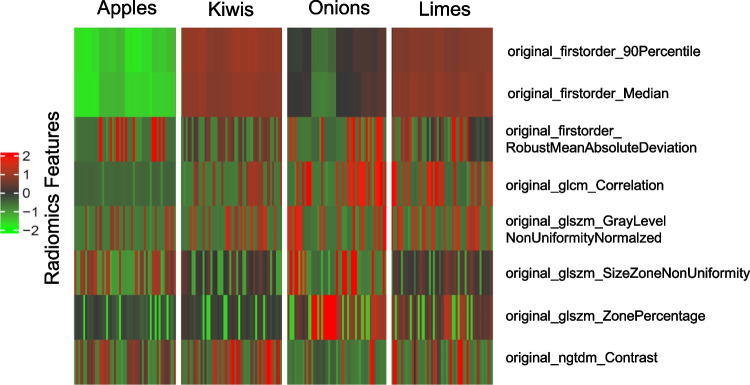
Inter-phantom stable features heatmap (ICC > 0.75)

The created phantom selective heatmap with all extracted radiomics features is shown in Fig. 5. The different phantom groups can be visually distinguished as individual clusters. Figure 6 shows a heatmap with the features determined in the ICC analyses (step III) that are stable over at least 3 phantom groups.

## Discussion

In this study, we demonstrated that the vast majority of the extracted radiomics features (70% of the parameters) have excellent test–retest stability on scans in the novel photon-counting CT. Several shape-based features and first-order features show a CCC value of > 0.999. *DependenceNonUniformity*, as a measurement of the similarity of dependence throughout the image, is the non-shape-based feature with the highest CCC value (0.99929).

In step III, 8 features could be identified, which were stable in 3 of 4 phantom groups, respectively (see Table 1). It shows that the selected first-order features in apples do not seem to be stable (possibly due to a larger individual deviation of shape and size within the apple group compared to the other phantom groups) as well as instability of all selected second-order features in onions. This may be explained by the multilayer structure of the onions with air between the individual layers, which could lead to larger differences in the calculation of the non-shape features. Given the varying feature stability measured as ICC between the phantom groups, an inherent phantom-related heterogeneity cannot be fully ruled out. Therefore, the overall feature stability may be underestimated.

To date, radiomics analysis has not yet been established in daily clinical practice due to the lack of comparability. In the past, multiple studies showed the influence of variable parameters on radiomics texture analysis. Zhao et al investigated the influence of different slice thicknesses on radiomics features using a chest phantom. In comparison to 5-mm slice thickness, 1.25- and 2.5-mm slice thicknesses were better suitable for homogeneity, volume, density means, and density SD gray level co-occurrence matrix (GLCM) energy. Additionally, the influence of different reconstruction algorithms on radiomics features was analyzed, outlining the standard reconstruction algorithms being best for density SD, whereas the lung reconstruction algorithms for density to mean, respectively [37].

In a phantom study by Jensen et al, radiomics features differed between ROI sizes and volume in both MRI and CT even though a homogenous phantom without any texture differences was used. Comparing both techniques, CT features were more stable than MRI features when tested for significance with the Mann–Whitney *U* test. On the other hand, OCCCs show excellent (> 0.90) agreement for different MR-derived features, but not for CT-derived features [38]. Mackin et al investigated the influence of sixteen different CT scanners, produced by four different manufacturers, and different acquisition parameters on a CT phantom in 2018. They outlined a changeability of radiomics features between the different scanners, suggesting that the comparability of radiomics studies depends on the consistency of image acquisition and reconstruction. Additionally, Mackin et al compared the radiomics features of a phantom with radiomics features derived from non-small cell lung cancer (NSCLC) tumors and proved comparability of variability in radiomics features between both groups [39].

Recently, Dunning et al scanned different organic samples (zucchini, onions, and oranges) on photon-counting detector CT (PCD-CT) and energy-integrating detector CT (EID-CT) at two dose levels. They calculated fourteen relevant radiomics features in each image for each sample and compared those between both scanners. Their results outlined a notable change by at least 50% of 13 out of 14 radiomics features at both dose levels by improved resolution of PCD-CT. Additionally, the higher Dunn Index of radiomics features derived from PCD-CT indicates better cluster separation for classification (Dunn Index 1.99 and 1.80 for PCD-CT and EID-CT respectively at 10 mGy and 2.44 (PCD-CT) and 1.65 (EID-CT) at 60 mGy), indicating an impact of high-resolution PCD-CT on radiomics analysis [30]. Our study goes one step further and outlines that this potential impact of high-resolution PCCT on radiomics analysis offers excellent test–retest stability on an organic phantom. In comparison, Peng et al detected stability of radiomics features in a test–retest setting between different time points on different conventional EID-CT in only 20.93% (CCC 0.56 ± 0.31) using an anthropomorphic thorax phantom consisting of three parts: body model, the internal structure of the lung, and simulated nodules. In further investigation steps, they compared the stability on different CT scanners and with varying image acquisition and reconstruction parameters including pitch, rotation time, tube voltage, tube current, FOV, slice thickness, reconstruction kernel, and iteration level. The tube current (mA/s) showed an ICC of 0.54 ± 0.32 (34.29%) [40]. Another study recently compared feature properties of non-scarred left ventricular myocardium between EICT and PCCT scanners. While mean and standard deviation derived from first-order features were mostly comparable between both detector types, higher-order features showed marked differences in mean and standard deviation, outlining the potential influence of the PCD through higher spatial resolution, better signal-to-noise ratio, and better detection of lower-energy photons on texture analysis [29]. In comparison to our study, this study did not focus on test–retest variability and not on intra-scanner comparability of radiomics features.

Varghese et al evaluated the reproducibility and robustness of computed tomography–based texture analysis metrics in 2021 using CT images of a customized 3D-printed texture phantom consisting of six different texture patterns. The 3D phantom was scanned on four CT scanners of different manufacturers to assess reproducibility. For robustness assessment, varying CT imaging parameters were used (slice thickness, field of view, tube voltage, and tube current). In total, 23.2% of the features showed excellent robustness and reproducibility (ICC ≥ 0.9) [41]. Even though our study shows better reproducibility, we used only one scanner and one varying image parameter (tube current), so further investigations on PCCT must follow.

The fact that the benefits of the novel technology of PCCT have already found their way into the clinical routine is already shown by a proof-of-concept study demonstrating that the spectral information in PCCT data sets can be used to help to detect and quantify anemia on contrast-enhanced scans [42] as well as preliminary studies outlining potential imaging biomarkers for arteriosclerosis in periaortic adipose tissue [12] and diffuse myocardial damage through coronary artery calcifications [13].

Our study also has several limitations. As a single-center study, no comparison of features between two different PCCT scanners was possible. Additionally, no analysis regarding feature stability between different types of scanners was obtained. Both issues should be addressed in further studies in the future to show a possible more stable feature analysis on PCCT in comparison to conventional EID-CT.

In conclusion, this study outlines the high stability of a large proportion of radiomics features using PCCT in a phantom model. In the past, radiomics analysis was hampered by the lack of comparability, which may be overcome by PCCT technology and therefore foster the implementation of a more stable radiomics analysis in clinical routine.

## Supplementary Information

Below is the link to the electronic supplementary material.

Supplementary file1 (PDF 80 KB)

## Abbreviations

CCC: Concordance correlation coefficient
CT: Computed tomography
EID: Energy-integrating detector
GLCM: Gray level co-occurrence matrix
GLDM: Gray level dependence matrix
GLRLM: Gray level run length matrix
GLSZM: Gray level size zone matrix
ICC: Intraclass correlation coefficient
MRI: Magnetic resonance imaging
NGTDM: Neighboring gray tone difference matrix
PCCT: Photon-counting computed tomography
PCD: Photon-counting detector
RF: Random forest
ROI: Region of interest
SD: Standard deviation

## Declarations

### Guarantor

The scientific guarantor of this publication is Dr. Isabelle Ayx.

### Statistics and biometry

One of the authors has significant statistical expertise.

### Methodology

• retrospective

• experimental

• performed at one institution
