# Supplementary material for: Phantom-based radiomics feature test–retest stability analysis on photon-counting detector CT
Source: Eur Radiol. 2023 Feb 21;33(7):4905–14. doi: 10.1007/s00330-023-09460-z (PMC10289937; doi:10.1007/s00330-023-09460-z)
Supplement: Supplementary file 1 — Supplementary file1 (PDF 80 KB) [file 330_2023_9460_MOESM1_ESM.pdf]

Feature	CCC
original_firstorder_10Percentile	0.9900362
original_firstorder_90Percentile	0.9994636
original_firstorder_Energy	0.9998979
original_firstorder_Entropy	0.9897289
original_firstorder_InterquartileRange	0.9949142
original_firstorder_Kurtosis	0.8140063
original_firstorder_Maximum	0.962495
original_firstorder_Mean	0.9971634
original_firstorder_MeanAbsoluteDeviation	0.7635346
original_firstorder_Median	0.9997334
original_firstorder_Minimum	0.8908763
original_firstorder_Range	0.9082322
original_firstorder_RobustMeanAbsoluteDeviation	0.9878893
original_firstorder_RootMeanSquared	0.9983531
original_firstorder_Skewness	0.9607822
original_firstorder_TotalEnergy	0.9998979
original_firstorder_Uniformity	0.9967396
original_firstorder_Variance	0.4199374
original_glcm_Autocorrelation	0.8196107
original_glcm_ClusterProminence	0.8976208
original_glcm_ClusterShade	0.8450552
original_glcm_ClusterTendency	0.8302495
original_glcm_Contrast	0.7075548
original_glcm_Correlation	0.9868904
original_glcm_DifferenceAverage	0.9511007

original_glcm_DifferenceEntropy	0.9767806
original_glcm_DifferenceVariance	0.548599
original_glcm_Id	0.9965811
original_glcm_Idm	0.9970362
original_glcm_Idmn	0.8765961
original_glcm_Idn	0.9805954
original_glcm_Imc1	0.9968669
original_glcm_Imc2	0.9973839
original_glcm_InverseVariance	0.9948286
original_glcm_JointAverage	0.819176
original_glcm_JointEnergy	0.9962394
original_glcm_JointEntropy	0.9945738
original_glcm_MaximumProbability	0.9980932
original_glcm_SumEntropy	0.9936386
original_glcm_SumSquares	0.742621
original_gldm_DependenceEntropy	0.9964564
original_gldm_DependenceNonUniformity	0.99929
original_gldm_DependenceNonUniformityNormalized	0.9980265
original_gldm_DependenceVariance	0.9971625
original_gldm_GrayLevelNonUniformity	0.9965805
original_gldm_GrayLevelVariance	0.4162592
original_gldm_HighGrayLevelEmphasis	0.818193
original_gldm_LargeDependenceEmphasis	0.9980113
original_gldm_LargeDependenceHighGrayLevelEmphasis	0.9871665
original_gldm_LargeDependenceLowGrayLevelEmphasis	0.9689217
original_gldm_LowGrayLevelEmphasis	0.6181235

original_gldm_SmallDependenceEmphasis	0.9617241
original_gldm_SmallDependenceHighGrayLevelEmphasis	0.9480671
original_gldm_SmallDependenceLowGrayLevelEmphasis	0.5740384
original_glrlm_GrayLevelNonUniformity	0.998518
original_glrlm_GrayLevelNonUniformityNormalized	0.9803993
original_glrlm_GrayLevelVariance	0.5860448
original_glrlm_HighGrayLevelRunEmphasis	0.7878003
original_glrlm_LongRunEmphasis	0.9959573
original_glrlm_LongRunHighGrayLevelEmphasis	0.9899075
original_glrlm_LongRunLowGrayLevelEmphasis	0.9755269
original_glrlm_LowGrayLevelRunEmphasis	0.6592198
original_glrlm_RunEntropy	0.9414159
original_glrlm_RunLengthNonUniformity	0.9990415
original_glrlm_RunLengthNonUniformityNormalized	0.9931074
original_glrlm_RunPercentage	0.9981576
original_glrlm_RunVariance	0.9957358
original_glrlm_ShortRunEmphasis	0.9919167
original_glrlm_ShortRunHighGrayLevelEmphasis	0.793669
original_glrlm_ShortRunLowGrayLevelEmphasis	0.6464164
original_glszm_GrayLevelNonUniformity	0.9983955
original_glszm_GrayLevelNonUniformityNormalized	0.7301771
original_glszm_GrayLevelVariance	0.5849947
original_glszm_HighGrayLevelZoneEmphasis	0.8420948
original_glszm_LargeAreaEmphasis	0.9361355
original_glszm_LargeAreaHighGrayLevelEmphasis	0.9531752
original_glszm_LargeAreaLowGrayLevelEmphasis	0.8496425

original_glszm_LowGrayLevelZoneEmphasis	0.6975779
original_glszm_SizeZoneNonUniformity	0.9776123
original_glszm_SizeZoneNonUniformityNormalized	0.9466885
original_glszm_SmallAreaEmphasis	0.9411288
original_glszm_SmallAreaHighGrayLevelEmphasis	0.7997679
original_glszm_SmallAreaLowGrayLevelEmphasis	0.7379672
original_glszm_ZoneEntropy	0.9231481
original_glszm_ZonePercentage	0.9669675
original_glszm_ZoneVariance	0.9364383
original_ngtdm_Busyness	0.9921699
original_ngtdm_Coarseness	0.9790965
original_ngtdm_Complexity	0.8351041
original_ngtdm_Contrast	0.7596957
original_ngtdm_Strength	0.9401035
original_shape_Flatness	0.9965128
original_shape_LeastAxisLength	0.9990963
original_shape_MajorAxisLength	0.9991669
original_shape_Maximum2DDiameterColumn	0.9489013
original_shape_Maximum2DDiameterRow	0.9985812
original_shape_Maximum2DDiameterSlice	0.9405163
original_shape_Maximum3DDiameter	0.9080986
original_shape_MeshVolume	0.999409
original_shape_MinorAxisLength	0.9989516
original_shape_Sphericity	0.9742998
original_shape_SurfaceArea	0.9993898
original_shape_SurfaceVolumeRatio	0.9990983

original_shape_VoxelVolume	0.9994095
**Number of features with CCC > 0.9**	**73**
	**70,19%**
